# Siloxane crosslinks with dynamic bond exchange enable shape programming in liquid-crystalline elastomers

**DOI:** 10.1038/s41598-020-63508-4

**Published:** 2020-04-20

**Authors:** Mohand O. Saed, Eugene M. Terentjev

**Affiliations:** Cavendish Laboratory, University of Cambridge, J. J. Thomson Avenue, Cambridge, CB3 0HE United Kingdom

**Keywords:** Polymers, Liquid crystals

## Abstract

Liquid crystalline elastomers (LCE) undergo reversible shape changes in response to stimuli, which enables a wide range of smart applications, in soft robotics, adhesive systems or biomedical medical devices. In this study, we introduce a new dynamic covalent chemistry based on siloxane equilibrium exchange into the LCE to enable processing (director alignment, remolding, and welding). Unlike the traditional siloxane based LCE, which were produced by reaction schemes with irreversible bonds (e.g. hydrosilylation), here we use a much more robust reaction (thiol-acrylate/thiol-ene ‘double-click’ chemistry) to obtain highly uniform dynamically crosslinked networks. Combining the siloxane crosslinker with click chemistry produces exchangeable LCE (xLCE) with tunable properties, low glass transition (−30 °C), controllable nematic to isotropic transition (33 to 70 °C), and a very high vitrification temperature (up to 250 °C). Accordingly, this class of dynamically crosslinked xLCE shows unprecedented thermal stability within the working temperature range (−50 to 140 °C), over many thermal actuation cycles without any creep. Finally, multiple xLCE sharing the same siloxane exchangeable bonds can be welded into single continuous structures to allow for composite materials that sequentially and reversibly undergo multiple phase transformations in different sections of the sample.

## Introduction

Liquid crystalline elastomers (LCE) have fascinated researchers for over 30 years, since the pioneering work has laid out the foundations of theoretical understanding^1,2^ and the materials chemistry^3^. The concept of a thermally-driven reversible actuation in LCE (artificial muscle)^4–6^ has been at the front of the search for practical applications^7^ ranging from sensors^8^ to soft robotics^9^ (although the unique ‘soft elasticity’ of LCE^10^ could promise an alternative route to applications in damping^11^ and adhesive systems^12^).

Traditionally, the principal methodology to prepare LCE actuators has been through the Finkelmann’s hydrosilylation reaction of siloxane monomers and vinyl or acrylate mesogens^3,4^. The alignment of an LCE by uniaxial stress^13^ (often called the polydomain-monodomain transition^14^) and the method of two-step crosslinking^15^ to produce the permanently aligned (monodomain) LCE capable of actuation formed the foundation of the field^2^.

The original work on LCE used siloxane-based elastomers as a material strategy due to their incredible properties (high failure strain, low glass transitions, and low moduli), attributed to the exceptionally flexible Si−O−Si linkages within the polymer backbone. However, it has proven to be problematic to achieve any useful configuration except the uniaxial alignment in flat film, due to the unavoidable limitations of two competing processes: orientation alignment and network crosslinking^15^.

Recently, the pioneering work of Leibler *et al*. introduced the concept of ‘vitrimers’ (polymer networks covalently crosslinked by a bond-exchange reaction)^16^. This appears to be a useful strategy to process LCE as well (i.e. solving the alignment problem)^17^. Vitrimers are much more stable than typical transient elastomer networks, allow full thermal re-molding (making the material renewable), and allow molding of complex shapes with intricate local alignment (which is impossible in traditional elastomers).

The first examples of such ‘exchangeable LCE’ (xLCE)^17^ were based on the transesterification bond-exchange reaction (BER)^17–19^, following the original work of Leibler *et al*.^16^. In the past few years, a number of strategies based on dynamic covalent bonds to achieve complex alignment in xLCE have been followed, such as disulfide^20,21^, free-radical addition fragmentation chain transfer^22^, exchangeable urethane bonds^23^, and more recently – the boronic transesterification^24^. All of these approaches are based on hydrocarbon elastomers and many of them share the problem of residual creep when the operating temperature of the elastomer is not sufficiently far from the activation temperature for the bond-exchange. This problem is often inherent because the hydrocarbon polymers and elastomers often have a high glass transition temperature, T_g_, so ‘operating temperature’ could be quite high as well, thus approaching the bond-exchange region.

Network plasticity in hydrocarbon elastomers with C-C bonds can be obtained in two ways: either through associative reactions (e.g. transesterification)^16^, where the network can alter its topology while upholding the constant number of covalent bonds, or via the bond cleavage and subsequent re-forming^25–27^ (i.e. dissociative reactions) in covalent adaptable networks (CAN). Examples of dissociative CAN reactions include Diels-Alder reaction^28^, or disulfide metathesis^29^.

Silicone-based elastomers (partially replacing carbon with silicone, which results in significant lowering of the glass transition) are an important class of polymers, widely used in low-temperature environment, often utilized as sealants, and in microfluidic fabrication due to their extreme hydrophobic nature and ideal mechanical properties. In principle, all of the methods used to add network plasticity to hydrocarbon-based elastomers can be incorporated in silicone-based elastomers as well. For example, vinylogous urethane exchange^30^, transesterification^31^, boroxine bonds^32^, or Meldrum’s acid-derived bonds^33^ were used with crosslinked polydimethylsiloxane (PDMS) systems. However, there are certain types of dynamic exchange unique to silicone-based bonds, such as the equilibrium exchange in the siloxane adaptable networks^34–36^, and the silyl ether metathesis^37^.

**Scheme 1:**
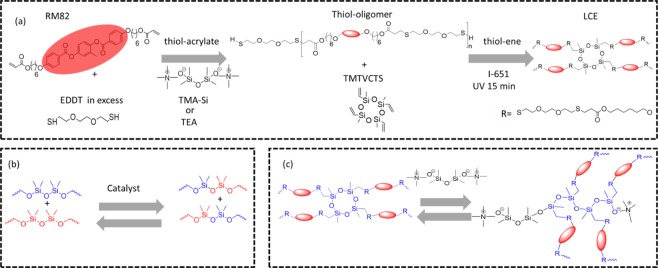
(**a**) The summary of ‘double-click’ chemistry: the mesogenic di-acrylate (RM82) first reacts with di-thiol (EDDT), which is in excess. Following that, the thiol-terminated oligomer chains are photo-polymerized with the vinyl bonds of the ring-siloxane, leading to the permanent network with 4-functional crosslinks. (**b**) The general scheme of siloxane exchange enabled by acid or base catalyst. (**b**) The principal route of siloxane exchange in our networks: the siloxanolate catalyst (TMA-Si) breaks the ring and terminates the linear 4-functional siloxane crosslink segment.

Here, we aim to ‘reinvent’ the use of siloxane in LCE systems, utilizing a much more robust ‘click’ chemistry to produce highly uniform crosslinked networks^38–40^. Firstly, we employ a two-stage, one-pot, thiol-acrylate/thiol-ene ‘double-click’ strategy to produce xLCE with tunable thermomechanical properties, see Scheme 1a. Secondly, we use the adaptable topology of a network with exchangeable siloxane bonds to impart the plastic flow and processability (i.e. the ability to re-program the alignment, re-mold, and re-shape the material, see Scheme 1c).

In this study, we select triethylamine (TEA) and tetramethylammonium siloxanolate (TMA-Si) to serve as catalysts for the thiol-acrylate Michael addition, and subsequently for the siloxane bond exchange. Unlike TEA, TMA-Si has a much higher evaporation temperature, and so it is expected to remain in the network even after prolonged exposure to high temperatures.

We identify the optimal processing conditions for the network alignment and plastic re-molding. These conditions serve as a guide to prepare programmed (permanently aligned, monodomain) elastomers, in a basic film and in complex shapes, and demonstrate the reversible thermal actuation of these new materials.

## Results and discussion

The main point of this paper is to introduce the new chemistry and processing approach: utilizing the ‘click chemistry’ based on thiols, employing thiols and siloxanes to control the glass transition, and benefitting from the equilibrium siloxane exchange to impart the dynamic adaptability to the resulting elastomers. There are broad opportunities in this approach: one is free to choose a number of different units from the existing library of di-acrylate reacting monomers^41^, and di-thiol chain extenders^42^, thus achieving different LC phases and properties for various practical aims and applications^43–51^.

The details of our formulations are given in the Experimental Section at the end. Here, it is important to introduce the notation: we characterize the material composition by the mol fraction of reacting bonds, thiol-acrylate and thiol-vinyl, always taking the content of mesogenic di-acrylate RM82 monomer as a 100% reference (or 1 molar ratio). Then our lowest crosslinking-density network, labelled as “x20” has 20% (or 0.2 molar ratio) of vinyl bonds on 4-functional ring-siloxane TMTVCTS crosslinks, and accordingly, the stoichiometric amount of 120% (or 1.2 molar ratio) of thiols on the di-functional chain extender EDDT, see Table 1.

**Table 1:** Chemical formulations and monomer mass (g) and mol function (mmol) of xLCE systems used. All systems are synthesized using thiol-acrylate/thiol-ene double click reaction.

Network description	Mass and mol functional of RM82 mesogen	Mass and mol functional of EDDT spacer	Mass and mol functional of TMTVCTS crosslinker
x20	1 g, 2.908 mmol	0.3341 g, 3.489 mmol	0.0511 g, 0.582 mmol
x40	1 g, 2.908 mmol	0.3898 g, 4.072 mmol	0.1023 g, 1.161 mmol
x60	1 g, 2.908 mmol	0.4454 g, 4.652 mmol	0.1534 g, 1.744 mmol
x80	1 g, 2.908 mmol	0.5011 g, 5.234 mmol	0.2045 g, 2.326 mmol
x100	1 g, 2.909 mmol	0.5568 g, 5.81 mmol	0.2557 g, 2.909 mmol

At the opposite end we have the highly crosslinked network, labelled as “x100”, which has 100% vinyl bonds on TMTVCTS crosslinks (1:1 with acrylate bonds of the mesogens), and accordingly 200% (or 2 molar ratio) of thiols. For instance, according to this nomenclature, the “x100” network has two RM82 mesogens per 4-functional crosslink, that is, on average network strands contain just one RM82 rod between two thiols. In the same way, the “x20” network has its strands, on average, with 5 RM82 rods separated by thiol spacers.

**Figure 1:**
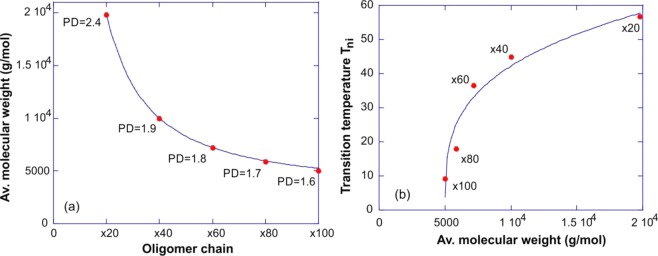
The effect of molecular weight of the oligomers: (**a**) Comparison of the average molecular weight (Mw) of thiol terminated oligomers, labelled in Table 1. The chain polydispersity PD is labelled on the plot for each data point. (**b**) The correlation between the nematic transition temperature Tni of thiol-terminated oligomers and their molecular weight Mw, with the network designation labelled for each data point. The power-law fit lines are a guide to the eye.

To verify the effect of our composition on the network strand length, we first characterized the molecular weight of thiol-terminated oligomer chains before adding the crosslinker (after the first, thiol-acrylate click reaction with an appropriate thiol excess, see Scheme 1). The oligomers are synthesized via the self-limiting thiol-acrylate Michael addition between RM82 and EDDT with molar ratio of 1.0:1.2 (x20), 1.0:1.4 (x40), 1.0:1.6 (x60), 1.0:1.8 (x80), and 1.0:2.0 (x100) (RM82: EDDT, respectively). Figure 1(a) shows the average molecular weight M_w_ of thiol terminated oligomers (obtained by gel permeation chromatography, GPC) for the formulations x20, x40, x60, x80 and x100 listed in Table 1. As expected, the average M_w_ of the LC chains decreases with the increasing molar ratio of the thiol monomer, which also acts to ‘dilute’ the mesogens and lower the nematic transition temperature. The chain polydispersity (PD) of these oligomers also decreases with increasing molar ratio of the thiol spacer, confirming that longer polymer chains also have higher polydispersity. More information about the molecular weight of the oligomers can be found in Table S1 of the Supporting information. The average chain length is expected to influence the phase behavior in LC oligomers, therefore, we used the differential scanning calorimetry (DSC) to determine the transition temperatures (Fig. S1, Supporting information). The nematic to isotropic transition (T_ni_) increases nonlinearly from 10 to 57 °C with the increasing chain length, Fig. 1(b).

**Figure 2:**
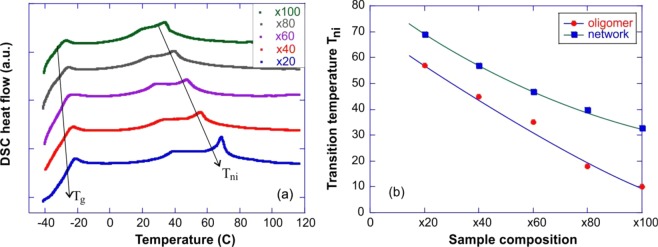
Differential scanning calorimetry (DSC) of our crosslinked networks (**a**), on heating, with the different crosslinking density labelled on the plot, showing the glass- and the nematic-isotropic transition temperature variation with composition. (**b**) Comparison of nematic to isotropic transition (Tni) of LC oligomers (without crosslinker) and LC networks after crosslinking.

Then these oligomers were crosslinked into elastomers by reacting them with siloxane vinyl crosslinker initiated under UV light (thiol-ene photopolymerization). The reaction conversion and the degree of polymerization were characterized via FTIR and gel fraction (Figs. S2 and S3, in the Supporting information). The DSC results of a series of crosslinked LCE networks enabled by varying molar ratio of siloxane vinyl crosslinker are shown in Fig. 2(a). The glass transition (T_g_) is around −30 °C, with very little changes even when the crosslinking density is significantly increased, which has to be attributed to flexibility of the siloxane crosslinker. On the other hand, the reduction of the molecular weight of the oligomers, which now become network strands, reduces the nematic-isotropic transition (T_ni_) after crosslinking, similar to what we saw in Fig. 1(b). This is an obvious effect of dilution of mesogenic units by the flexible thiol spacers and siloxane crosslinkers. This comparison is summarized in Fig. 2(b), also showing that the crosslinking markedly increases the intrinsic mesogenic power of the material (by reducing mobility of flexible terminals of oligomers). Note, that even the x100 LCE network has a broad range of the liquid-crystalline phase below T_ni_~33 °C. T_ni_ increases by ~ 12° after crosslinking for compositions of x20, x40, and x60 compared to ~22° for compositions of x80 and x100.

**Figure 3:**
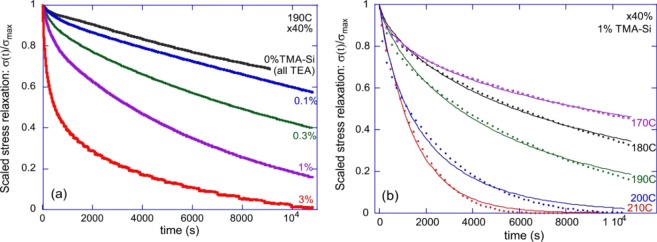
(**a**) Scaled stress-relaxation σ(t)/σ_max_ for the x40 LCE network at T = 190 °C, for several concentrations of catalyst, as labelled in the plot. (**b**) Stress relaxation curves for 1 wt% of TMA-Si, at several temperatures labelled in the plot. Dashed lines are the fits with the exponential relaxation function, which produce the relaxation time τ, which is a sharp function of temperature.

Figure 3 shows the results of a typical stress-relaxation in the siloxane-based xLCE, which takes place after an instant fixed-strain is imposed on the sample (maintaining the constant temperature). The results are presented via a scaled relaxation function σ(t)/σ_max_, in order to focus purely on the time dependence. The normalized stress as function of time for x40 samples containing various TEA and TMA-Si concentrations is shown in Fig. 3(a). We compare the networks having a fixed total of 1 wt% of a catalyst mixture of TMA-Si and TEA, in ratios 1:0, 0.3:0.7, 0.1:0.9 and 0:1 between the two, respectively. The slowest relaxation is in the sample with 1 wt% TEA (labelled as 0% TMA-Si in the plot); the increasing fraction of TMA-Si makes the bond exchange faster. Both of these amines can trigger the relaxation of the siloxane elastomer, however, TEA is a more volatile catalyst at elevated temperatures. Therefore, it generates slower stress relaxation compared to TMA-Si. For comparison, we also show the relaxation of an xLCE with 3 wt% of TMA-Si catalyst, which predictably is much faster.

**Figure 4:**
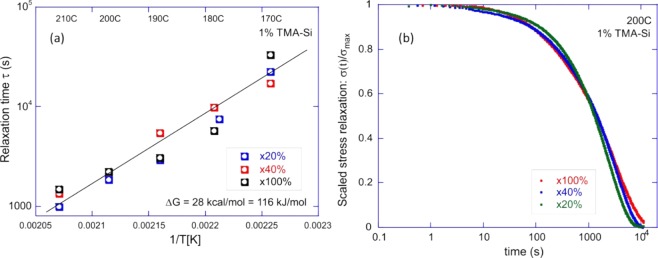
(**a**) The Arrhenius plots for the relaxation time τ(T), for different xLCE networks. The slope of the linear fitting gives the bond strength ΔG≈28 kcal/mol, and the additive constant gives the ‘rate of attempts’ ω_0_. Three data sets are for the x20, x40 and x100 networks, all fit with the same activation law. (**b**) Comparison of the scaled stress relaxation at 200^o^ C for the same x20, x40 and x100 networks, illustrating that the rate of relaxation is the same for all. The logarithmic time axis helps the comparison simultaneously at short and long times.

The fitting of such scaled stress relaxation curves with the basic exponential relaxation, for 1 wt% TMA-Si is shown in Fig. 3(b). It gives the characteristic relaxation time τ(T) for each material and temperature. As expected, increasing the temperature accelerated the relaxation, where at 210 °C the elastomer is fully relaxed after 7000 s due to its internal plastic flow. To study the influence of the siloxane concentration on the stress relaxation, we tested siloxane crosslinked networks containing various siloxane concentrations (e.g. x20, x40, and x100), each network having the same amount of catalyst (1 wt% of TMA-Si). The data of the relaxation times for various samples were then collated at different temperatures to generate the Arrhenius plot, Fig. 4(a). That is, we plot τ(T) on the logarithmic scale, and fit the data with the thermal activation law: τ =ω_0_^−1^ ∙exp(ΔG/k_B_T), or log[τ] = const + ΔG/k_B_T.

It is clear that data sets show a single value of activation energy ΔG ≈28 kcal/mol (or 116 kJ/mol), which corresponds to about 45 k_B_T at room temperature, and is in good agreement with the results of Xie *et al*.^52^ who used 0.1 wt% of sodium octanoate as catalyst in a much higher siloxane concentration elastomer (Sylgard 184 PDMS). In comparison, in the work of Leibler *et al*.^16^, the transesterification with the zinc acetate catalyst had the activation energy ΔG ≈20 kcal/mol (or 34 k_B_T). It is expected, and reassuring, that the single value of activation energy ΔG describes the macroscopic stress/relaxation: this is a clear signature of a distinct reaction pathway, in our case depicted in Scheme 1c.

Surprisingly, siloxane elastomers with very different concentration of crosslinker appear to have the same ‘rate of attempts’ ω_0_ in their relaxation behavior. It is confirmed by comparing the relaxation curves themselves for these different networks at the same temperature in Fig. 4(b). To explain this, we argue that the exchange route in Scheme 1c (the siloxane ring opening) is the dominant route for the bond exchange, and for the relatively low siloxane fraction in the network the overall rate is limited by the catalyst concentration, rather than the amount of reacting sites.

**Figure 5:**
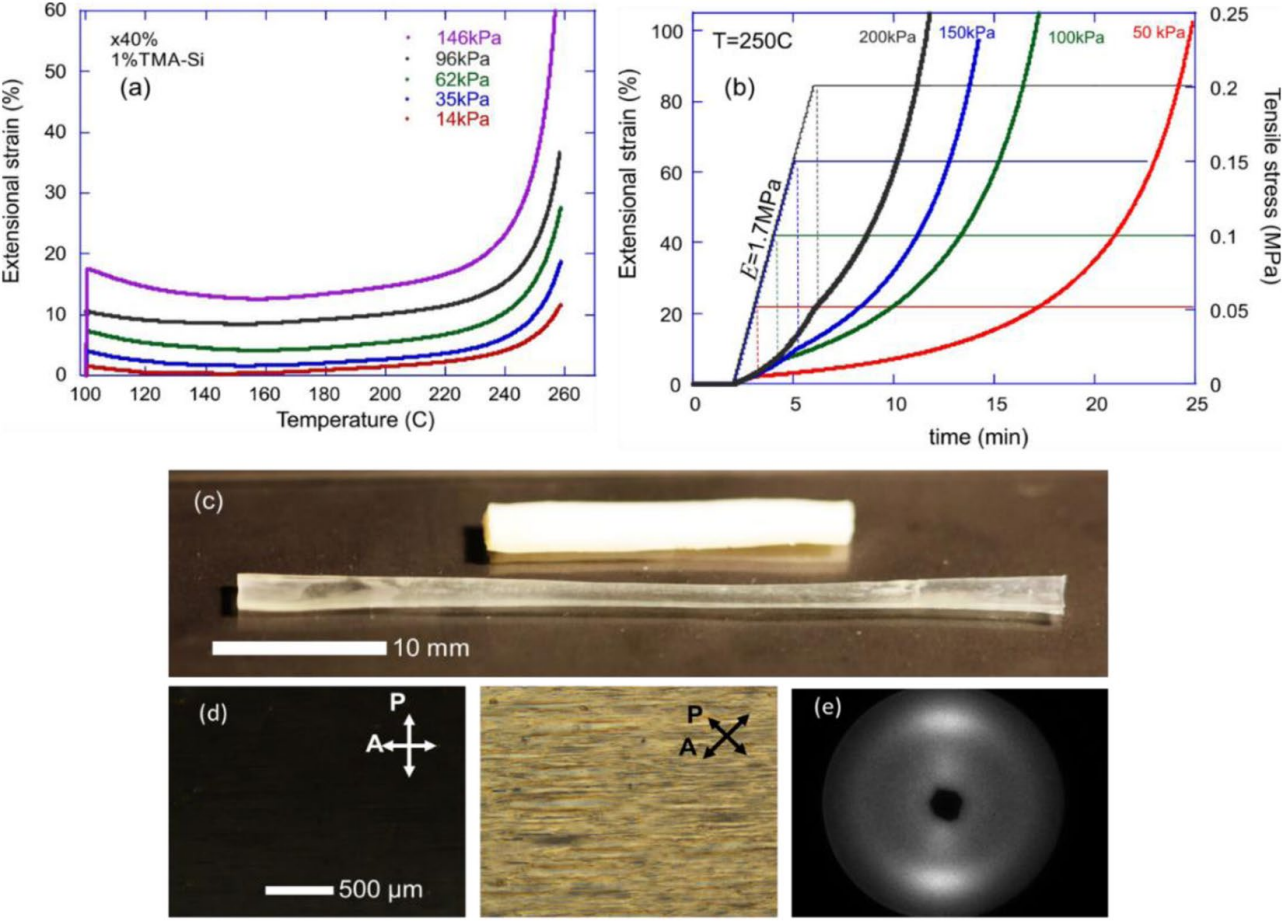
(**a**) The ‘iso-stress’ curves show the strain changing with temperature at constant stress (labelled on the plot). The equilibrium rubber modulus of the x40 xLCE at 100 °C (in the isotropic phase) is E = 880 kPa, which gives the initial strain in all curves. At temperatures below T_v_ we see the classical effect of entropic elasticity, the rubber modulus linearly increasing with T. The onset of plastic flow occurs around 140–160 °C, with the network in free-flow above 250 °C. (**b**) Programming of the aligned monodomain in xLCE. With a sample kept at constant T = 250 °C (resulting in a higher rubber modulus E), we apply a tensile stress (labelled on the plot), and allow the plastic flow to reach 100% extension, at which point we consider the structure aligned, and take the sample out of the device. The time for this programming is several minutes, depending on the applied tensile load. (**c**) The initial polydomain and uniaxially aligned monodomain xLCE, programmed by its plastic flow to 100% elongtion. (**d**) The microscopy images between crossed polars indications alignment. (**e**) X-ray image confirming a good uniaxial nematic alignment, also letting us calculate the order parameter Q = 0.62.

Figure 5 illustrates the dynamic response of our xLCE, due to the siloxane exchange reaction allowing plastic flow under stress, at a sufficiently high temperature. First, we study the ‘iso-stress’ response on changing temperature, which is often incorrectly called ‘dilatometry’ in the literature. Dilatometry is a valid test of changing the sample volume, while in these elastomer networks the volume is certainly constant. What we test is how the uniaxial extension changes with temperature in the sample under constant tensile stress, Fig. 5(a). It is not an easy experiment in an LCE material because the effect of LCE thermal actuation produces a massive strain change on heating into the isotropic phase. Here we are concerned with the elastic-plastic transition of the exchangeable network, and so we start at T = 100 °C, well in the isotropic phase (the x40 network is chosen for this demonstration). First, we apply a given stress (as labelled in the plot), and register the resulting extensional strain, which gives the value of the Young modulus of the material (E ≈ 880 kPa in the 40% crosslinked xLCE), and then increase the temperature at a constant rate of 2°/min.

The first observation one makes is the classical rubber-elastic response: as the entropic rubber modulus increases with temperature, at constant stress the strain decreases^53^. The presence of this rubber-elastic region is another proof of thermal stability of our siloxane-crosslinked elastomers: in many less stable dynamically crosslinked network one does not find this region clearly identified, and strain increases (creep occurs) much below the full elastic-plastic transition. However, as the temperature increases further, and the bond-exchange becomes more prominent, the plastic flow (creep) starts being noticeable. The region where the data deviates from the initial rubber-elastic decreasing slope is the onset of the transition to plastic flow, or the vitrification point T_v_: apparently it does not depend on the applied stress^54^. One should expect some creep under stress in networks with siloxane-exchange above 140–150 °C, although the rapid flow only sets in at a much higher temperature (above 250 °C).

We use this regime of stress-induced plastic flow to program our xLCE materials into monodomain aligned state. Figure 5(b) illustrates the process: we bring the sample to a high temperature (T = 250 °C) as suggested by the results of iso-stress test, then apply a constant tensile stress to a level labelled in the plot, and then keep the sample at these constant temperature and stress until its elongation reaches 100% (clearly, this happens faster at higher stress, but in all cases the process takes several minutes and allows easy control). We deem the 100% elongation to be sufficient to impart the fully uniaxial monodomain alignment to our xLCE, and then take the programmed sample off the stress and heating. Figure 5(c–e) illustrates the aligned sample (comparing it with the initial polydomain xLCE also shown in Fig. 5(c). This programmed alignment is permanent as long as we do not allow the sample temperature to raise above 140 °C, cf. Fig. 5(a), when the residual creep would cause a gradual loss of alignment (increasing at even higher temperatures). However, as with all exchangeable xLCE systems, we can re-program the material to a different shape and state of alignment by a subsequent process.

**Figure 6:**
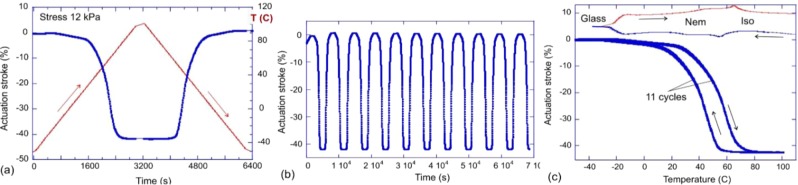
(**a**) One cycle of heating-cooling of the x40 xLCE network, with the temperature on the right y-axis, and the associated sample strain showing the classical reversible thermal actuation of LCE. (**b**) The cyclic contraction-extension during 11 heating cycles. (**c**) The actuation strain plotted against temperature, showing the reproducibility of actuation, and also the extent of thermal hysteresis at the heating rate of 3°/min applied in this test.

**Figure 7:**
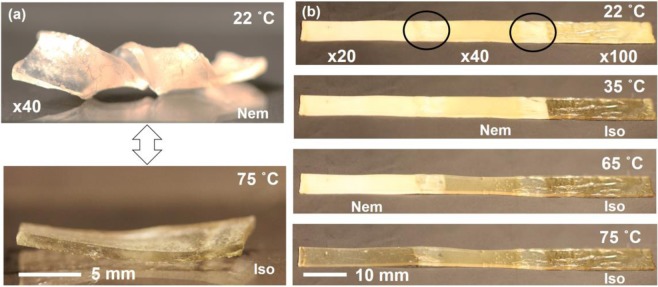
(**a**) The programmed shape of a helix, reversibly unwinds into a flat strip in the isotropic phase. (**b**) The thermally molded continuous strip combining three different xLCE materials: x20, x40, and x100, cf Table 1, molded together. Since the nematic-isotropic transition in each material is at a different temperature, we see different sections of the sample become sequentially isotropic on heating.

Having programmed the uniaxial monodomain alignment in xLCE, we can now examine its actuation response to reversible heating and cooling through the nematic-isotropic transition. Figure 6 illustrates different elements of this test, carried out in a DMA instrument under a low constant stress (of 12 kPa) applied to ensure the sample is straight and taut. In Fig. 6(a) we zoom-in on one cycle of heating and cooling, over the range of −50 °C to 90 °C (the x40 network is used, with its T_g_ ≈ −20 °C and T_ni_ ≈ 60 °C). The sample starts rapid contraction when the temperatures approaches 30 °C, and reaches the saturation strain of over 40% at around 70 °C (both values are clearly affected by the dynamics of heat exchange). On cooling the cycle reverses, and Fig. 6(b) illustrates the remarkable stability of this spontaneous contraction-expansion over 11 cycles of temperature. We expect no creep of thermal degradation to occur in our xLCE materials as the temperature never reached the levels where plastic effects might set in. The same 11 cycles of heating and cooling are shown in Fig. 6(c) as actuation strain against temperature: all heating and all cooling strokes are precisely on top of each other, however, we also notice a clear hysteresis of the nematic-isotropic transition. To support this observation, Fig. 6(c) also shows the DSC scans (scaled, in a.u.) on heating and cooling, at the top of the plot, to illustrate where the glass and nematic transitions are. The wide separation of the nematic transition and the vitrification temperature, at which the plastic creep starts to occur in the xLCE under stress, is the reason for stability of the thermal actuation and the programmed alignment pattern. The second remarkable aspect of xLCE (and indeed all vitrimers) is their capacity of thermal molding. We demonstrate this capacity on the following example: we take three different xLCE materials: with x20, x40, and x100 networks (cf. Table 1). Three strips are molded together into one continuous sample, at T = 250 °C and high pressure, kept overnight. It is reassuring that the thiol-siloxane mesogenic system has such a remarkable thermal stability (few polymers will withstand several hours at 250 °C without any degradation). Figure 7 illustrates the result of this molding, where one cannot distinguish the initial overlap regions. In this photo, at room temperature, all three sections are in the polydomain nematic state, and so white (strongly scattering light). Then, on heating this strip, we see the sequential phase transitions into the isotropic phase that take place in different sections of the otherwise continuous polymer strip: first the x100 section/becomes isotropic (transparent, no longer scattering light), then the x40 section, until finally the whole strip becomes isotropic.

This paper presents a new material concept, and its characterization, so we did not aim to construct complex actuating shapes – merely to demonstrate the capacity to mold together different xLCE materials containing exchangeable siloxane bonds and the appropriate catalyst. This offers rich design options for complicated actuating shapes and constructions for practical applications.

## Conclusions

In summary, we have designed a new class of exchangeable LCE network using a robust click chemistry (‘double click’ of thiol-acrylate and thiol-ene) and utilized the siloxane segments in the crosslinkers. These materials have several important advantages over the previous generations of LCE, which also use the commercial off-the-shelf starting ingredients: [1] the presence of thiols and siloxanes makes the glass transition naturally low; [2] they allow good control of the nematic transition, including bringing the T_ni_ down to the ‘human range’ of 30–40 °C that allows control of actuation by body heat; [3] the siloxane bond-exchange reaction imparts the dynamic network properties, similar to vitrimers: the plastic flow under stress at a high temperature allows both the programming of monodomain textures in the xLCE, and the (re)molding of plastic samples into desired structures. Obviously, the use of siloxane in the LCE is not limited to the crosslinkers, as in this paper: main-chain LCE with siloxane spacers, and side-chain LCE with siloxane backbone are well known. In these systems, like in our work here, the siloxane bond-exchange would be equally possible and useful. While revising this work, a paper disclosing the same reaction strategy has been published by Ji and co-workers^55^. These authors used siloxane spacers (as opposed to our crosslinks), and the same TMA-Si catalyst (following McCarthy *et al*.^36^, which in turn followed Grubb *et al*.^35^) – and obtained a smectic LC phase due to the micro-phase separation of siloxane spacers and mesogens, but with the same capability to re-mold and re-program the xLCE.

## Experimental Section

### Materials

Acrylate liquid crystal (LC) monomer, RM82 was purchased from Wilshire Technologies, Inc. Thiol chain extender, EDDT and vinyl siloxane crosslinker, 2,4,6,8-Tetramethyl-2,4,6,8-tetravinyl cyclotetrasiloxane (TMTVCTS), were purchased from Sigma-Aldrich. Tetramethulammonium siloxanolate, TMA-Si was purchased from Gelest and used as Michael addition base-catalyst and as anionic initiator to the siloxane bond exchange. Triethylamine (TEA) was purchased from Sigma-Aldrich and used as base-catalyst. The photoinitiator, Irgacure I-651, purchased from Sigma-Aldrich. Toluene and Tetrahydrofuran, THF were purchased from Sigma-Aldrich and used as solvent.

### Preparation of networks

We took advantage of the thiol reaction selectivity towards different functional groups to design one pot two-step thiol-acrylate/thiol-ene reaction sequence to prepare LCE with controllable nematic transition temperatures from the commercially available starting materials. We first prepare LC oligomers via the self-limiting thiol-acrylate Michael addition between a mesogenic diacrylate (RM82) and an isotropic dithiol (EDDT). The Michael addition was catalyzed via TMA-Si or TEA. By controlling the molar ratio of thiol to acrylate, thiol-terminated oligomers were obtained. The di-thiol oligomer is radically crosslinked with vinyl siloxane crosslinker, TMTVCTS. The overall reaction scheme is similar to a previously reported method^44^. In a 25 ml vial the intended amount of catalyst TMA-Si (0.1, 0.3, 1, or 3 wt%), was initially dissolved in a mixture of solvent (20 wt% THF and 20 wt% toluene), to this solution, RM82 was added and heated to 80 °C until fully dissolving. After the mixture was cooled down to room temperature, I-651 (1.5 wt%), EDDT, and TMTVCTS were added and mixed vigorously using vortex mixer. The monomers solution was degassed using a vacuum chamber and then quickly transferred into a mold (two glass sides with 1 mm spacer coated with Rain-X, anti-sticking agent). The monomer mixture was kept at 50 °C to fully oligomerize via Michael addition reaction for 12 h. Then the thiol-terminated oligomer was photopolymerized with TMTVCTS via 365 nm UV light for 15 min at 50 °C. The molar ratio used was 1.0 acrylate:1.4 thiol:0.4 vinyl, unless otherwise noted. After the polymerization was done, the samples were removed from the mold and placed in a vacuum oven at 80 ^o^C for 12 h to remove the solvents.

### Molecular weight of oligomer

The molecular weight of the oligomers was characterized with gel permeation chromatography (GPC: JASCO PU-98). The oligomers were synthesized via the self-limiting thiol-acrylate Michael addition between a nematic diacrylate (RM82) and an isotropic dithiol (EDDT) with molar ratio of 1.0:1.2, 1.0:1.4, 1.0:1.6, 1.0:1.8, and 1.0:2.0 (acrylate: thiol, respectively). The oligomers were first dissolve in DMF and then injected to the GPC (5 ml). All GPC tests were performed at 40 °C.

### Differential scanning calorimetry (DSC)

DSC4000 PerkinElmer was used to obtain the transition temperatures. Samples with ≈10 mg were loaded into standard aluminum DSC pans. The samples were heated to 120 °C at 10°min^−1^, held isothermally for 5 min to undo the thermal history, and cooled to −50 °C at 10°min^−1^. Then samples were heated again to 120 °C to obtain the data. T_g_ could be found at the step change in the slope of the heat flow signal and T_ni_ could be obtained at local minimum of the endothermic peak. The sample was run three times.

### Stress relaxation measurements

DMAQ800 (TA instruments) was used to characterize the relaxation behavior of siloxane crosslinked LCE. Samples with dimensions of ≈15 mm × 5 mm × 0.9 mm were tested. All of the samples were tested under constant uniaxial strain 3% imposed at t = 0, the strain was held constant isothermally for 180 min at 170, 180, 190, 200, or 210 °C. Prior imposing the strain, samples were kept at the desired temperature for 5 min. Samples were annealed at 80 °C for 12 h before the relaxation test.

### Iso-force measurements

DMAQ800 (TA instruments) was used to characterize the plastic flow of siloxane crosslinked LCE induced by siloxane bond exchange as a function of temperature. Samples with dimensions of ≈15 mm × 5 mm × 0.9 mm were tested. All of the samples were tested under constant uniaxial stress of 14, 35, 65, 96, 0r 146 kPa imposed at t = 0, the stress was held constant while the temperature was ramped at 2 ^o^C/min until 260 °C. Prior imposing the stress, samples were kept at the desired temperature for 5 min. Samples were annealed at 80 °C for 12 h before the relaxation test.

### Programing monodomain measurements

DMAQ800 (TA instruments) was used to align polydomain samples into monodomain via creep test. Samples with dimensions of ≈15 mm × 5 mm × 0.9 mm were tested. All samples were tested under constant uniaxial stress of 50, 100, 150, or 200 kPa imposed at t = 0, the stress was held constant isothermally at 250 °C until the strain reached 100%. Prior imposing the stress, samples were kept at the desired temperature for 2 min. After reaching 100% strain the samples were kept starched while cooling to room temperature. Samples were annealed at 80 °C for 12 h before the relaxation test.

### Wide angle x-ray scattering (WAXS)

The phase of the monodomain LCE at room temperature was characterized using a Philips diffractometer (PW-2233/20) with the wavelength of 0.154 nm, and the area detection CCD camera Gemstar 2 (Photonic Sciences). The beam size was ~ 0.7 × 0.7 mm^2^ with a flux of 4 × 10^9^ photons/s. The distance between the sample and the imaging area was 100 mm. The sample (0.5 mm × 6.5 mm and 20 mm) was exposed to the X-ray source for 20 seconds.

### Actuation measurements

Discovery DMA850 (TA instruments) was used to measure the actuation performance for the monodomain film. Rectangular samples measuring approximately 15 mm × 5 mm × 0.5 mm were tested in tensile mode. To measure actuation strain, a constant stress (12 kPa) was applied to the LCE film; each sample was heated and cooled at least 11 times from 100 to −50 °C, at 3° min^−1^.

### Welding conditions

Moore hydraulic press (Birmingham, England) was used to hot press the LCE samples. Samples (x20, x40, and x100) were first held at 250 ^o^C for 15 min before applying a load on 0.5 ton. The samples were allowed to cool (overnight) to room temperature under the applied load.

### The table of contents entry

Liquid crystalline elastomers with siloxane dynamic exchangable bonds are designed using a robust ‘double click’ chemistry of thiol-acrylate and thiol-ene, and utilizing siloxane segments in the crosslinkers. The plastic flow is used to process and program the xLCE materials (induce monodomain alignment, weld, and remold). The resulting aligned monodomain xLCE networks demonstrate large reversible thermal actuation.

## Supplementary information

Supplementary information.
