# Supplementary material for: Siloxane crosslinks with dynamic bond exchange enable shape programming in liquid-crystalline elastomers
Source: Sci Rep. 2020 Apr 20;10:6609. doi: 10.1038/s41598-020-63508-4 (PMC7171139; doi:10.1038/s41598-020-63508-4)
Supplement: Supplementary file 1 — Supplementary information. [file 41598_2020_63508_MOESM1_ESM.docx]

Supporting Information

Siloxane crosslinks with dynamic bond exchange enable shape programming in liquid-crystalline elastomers

Mohand O. Saed, Eugene M. Terentjev

Cavendish Laboratory, University of Cambridge, Cambridge CB3 0HE, U. K.

**Table 1**. The molecular weight of the oligomers was characterized via gel permeation chromatography (GPC). The oligomers were synthesized via the self-limiting thiol-acrylate Michael addition between a nematic diacrylate (RM82) and an isotropic dithiol (EDDT) with molar ratio of 1.0:1.2 (x20), 1.0:1.4 (x40), 1.0:1.6 (x60), 1.0:1.8 (x80), and 1.0:2.0 (x100) (acrylate: thiol, respectively).

| Thiol oligomer | M_n_  [g/mol] | M_w_ [g/mol] | M_z_  [g/mol] | D | Vp  [mL] | Mp [g/mol] |
| --- | --- | --- | --- | --- | --- | --- |
| 20x | 8.23E+03 | 1.98E+04 | 3.96E+04 | 2.4073 | 30.667 | 1.55E+04 |
| 40x | 5.32E+03 | 1.00E+04 | 1.60E+04 | 1.8803 | 32.142 | 9.09E+03 |
| 60x | 4.10E+03 | 7.20E+03 | 1.12E+04 | 1.7748 | 32.950 | 6.66E+03 |
| 80x | 3.42E+03 | 5.86E+03 | 9.19E+03 | 1.738 | 33.867 | 4.56E+03 |
| 100x | 3.09E+03 | 5.03E+03 | 7.71E+03 | 1.6258 | 35.358 | 2.29E+03 |


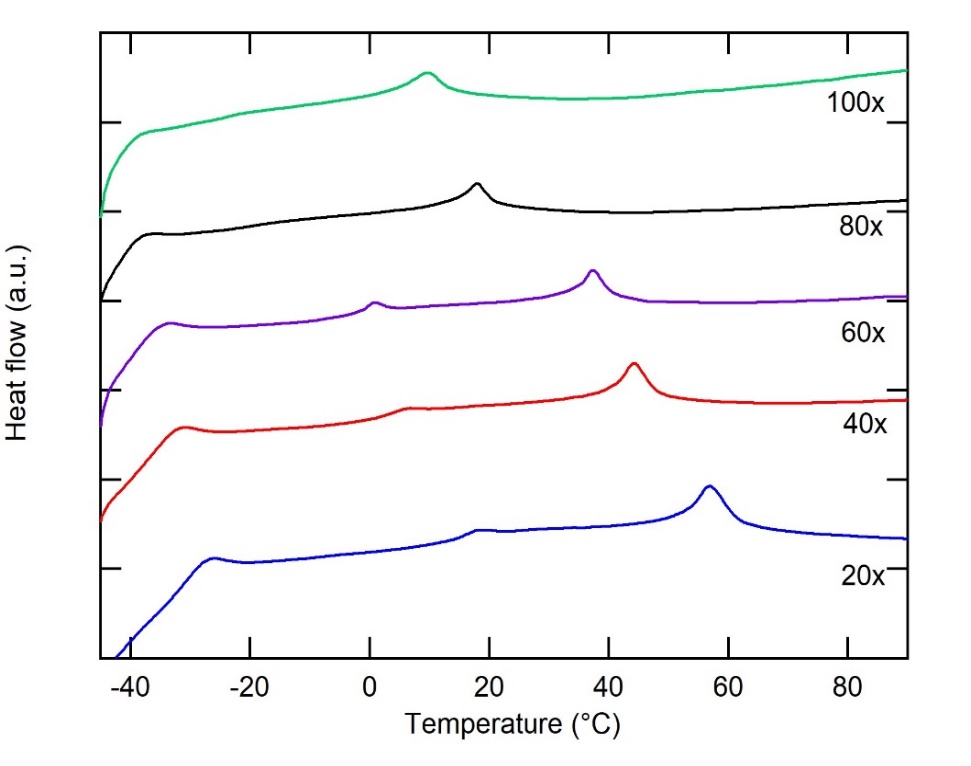


**Figure S1.** The liquid crystalline transition temperatures of the oligomers were characterized via differential scanning calorimetry (DSC). The oligomers were synthesized via the self-limiting thiol-acrylate Michael addition between a nematic diacrylate (RM82) and an isotropic dithiol (EDDT) with molar ratio of 1.0:1.2 (x20), 1.0:1.4 (x40), 1.0:1.6 (x60), 1.0:1.8 (x80), and 1.0:2.0 (x100) (acrylate: thiol, respectively).

As an example, we used x40 formulation to study the reactions conversion of the first stage reaction (thiol-acrylate catalyized via TMA-Si) and the second stage reaction (thiol-ene photo polymerization) using FTIR. The thiol, acrylate, and vinyl functional group conversions versus time (Figure S2). Conditions: stoichiometry of thiol: acrylate and vinyl is used, (1.4: 1.0:0.4 molar ratio for thiol: acrylate: vinyl, respectively). Thiol-acrylate reaction was carried out for 17h using TMA-Si as a catalyst. Complete disappearance of the acrylate peak (at 812 cm^-1^) occurred after 4 hr, while there was a present of thiol peak at 2571 cm^-1^. The thiol terminated oligomer was subsequently reacted with the siloxane vinyl crosslinker via thiol-ene reaction through UV for 10 min. Both reactions were carried out in solvent at 50˚C. The get fraction of the resulted elastomer is 94 ± 2 % (Figure S3, supporting information).


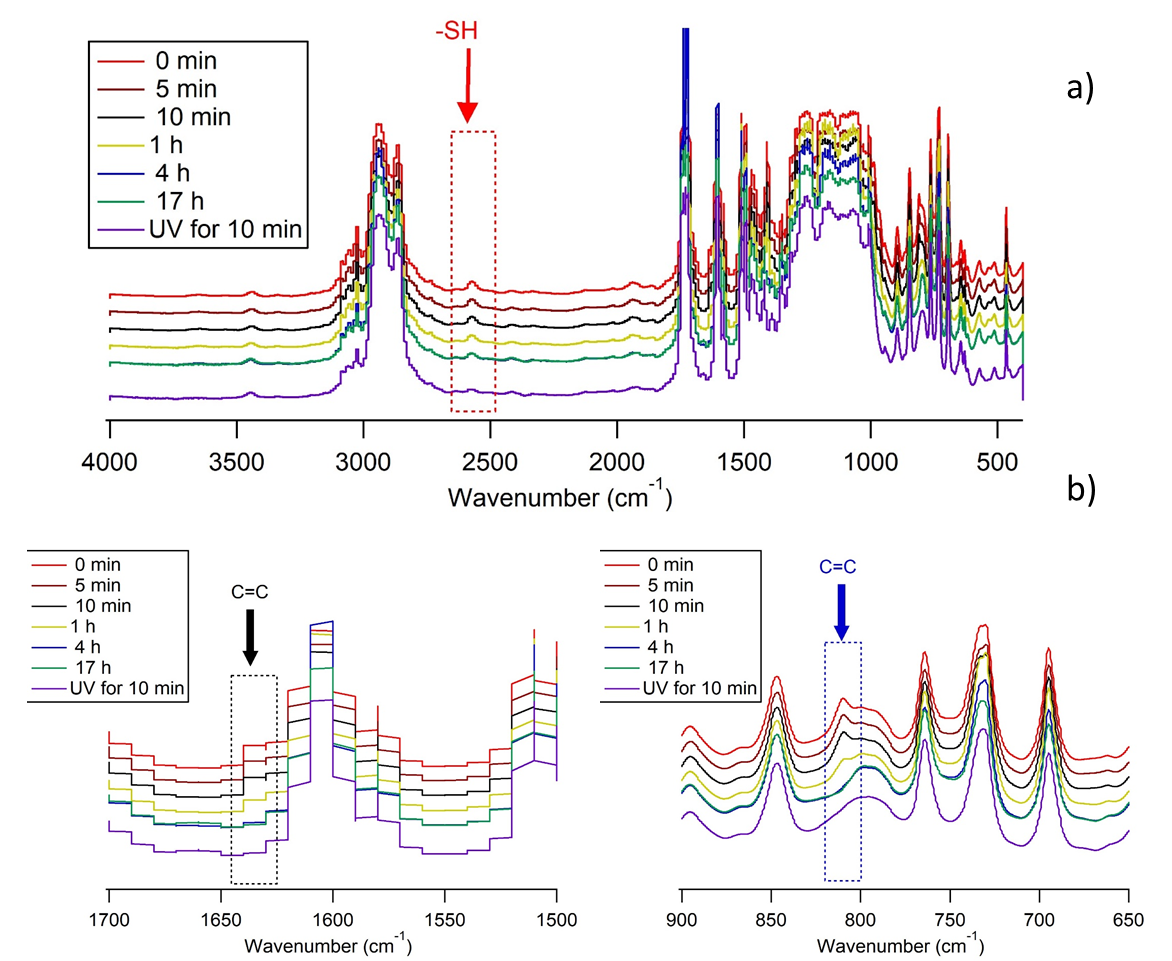


**Figure S2*.*** FTIR studies were conducted using a Nicolet 750 Magna FTIR spectrometer with a KBr beam splitter and an MCT/A detector. Series scans were recorded, taking spectra at the rate of approximately 2 scans per second. The thiol, acrylate, and vinyl functional group conversions versus time. Conditions: stoichiometry of thiol: acrylate and vinyl is used, (1.4: 1.0:0.4 molar ratio for thiol: acrylate: vinyl, respectively). Thiol-acrylate reaction was carried out for 17h using TMA-Si as a catalyst and thiol-vinyl reaction was triggered via UV for 10 min after the completion of the acrylate groups. Both reactions were carried out at 50˚C. a) Shows the disappearance of thiol peak (2571 cm^-1^) over time, b) shows the disappearance of the acrylate any vinyl peaks at (1636 cm^-1^ or 812 cm^-1^).


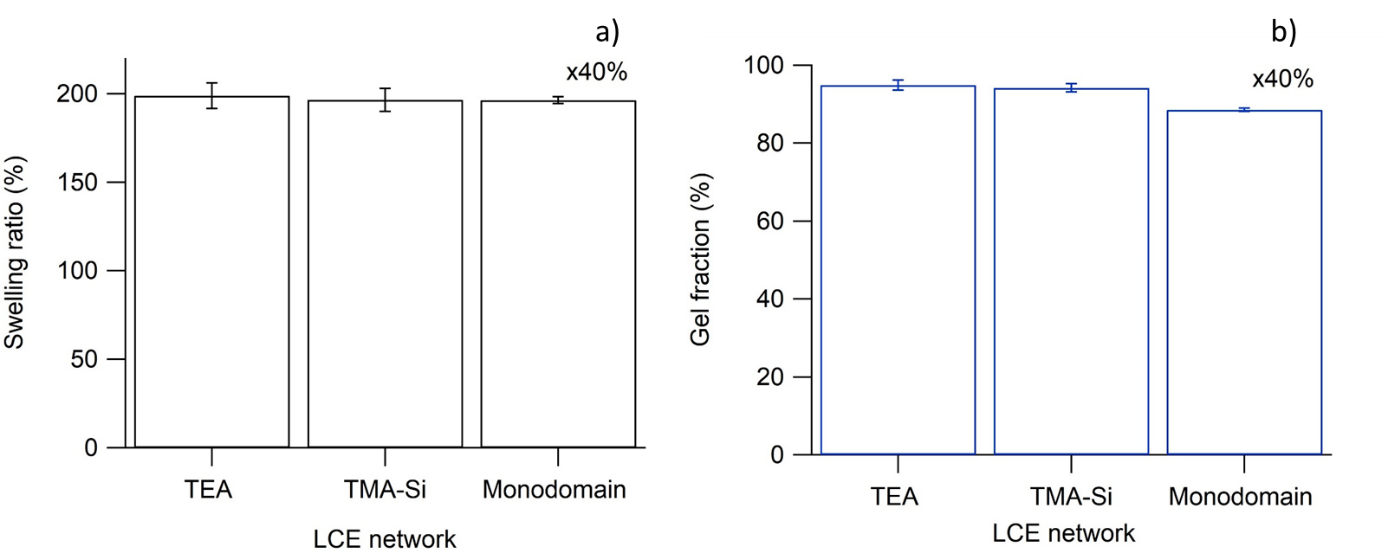


**Figure S3.** Swelling and gel fraction experiments were performed to determine the swelling ratio and gel fraction of LCE samples. The test is done for the x40 samples containing 40% mol fraction crosslinking (e.g. 1.4: 1.0:0.4 molar ratio for thiol: acrylate: vinyl, respectively). Three batches of the LCE formulation were synthesized using 1 wt% TEA (polydomain), 1 wt% TMA-Si (polydomain), and 1 wt% TMA-Si (monodomain). The monodomain samples were programed at 250˚C for 20 min). a) Swelling ratio (~200%) of samples after placed in toluene for 72 h at room temperature, b) gel fraction (~92 to 94%) for samples after drying the toluene under vacuum at 120˚C for 12 h. Three samples were tested for each network.


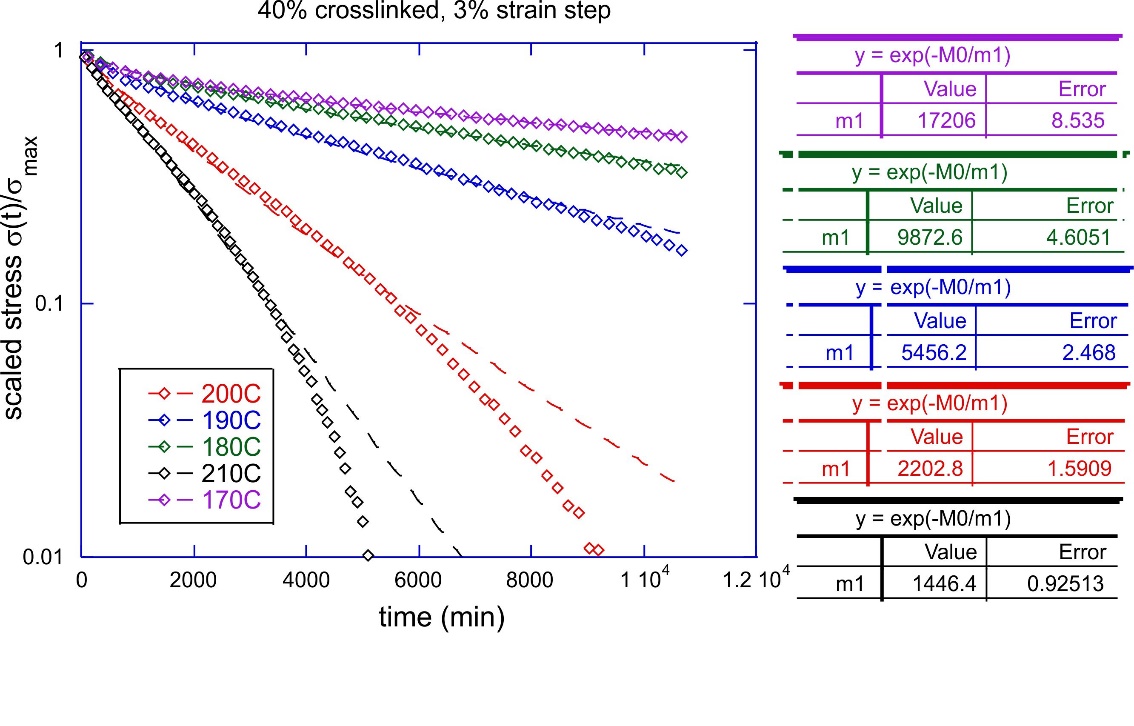


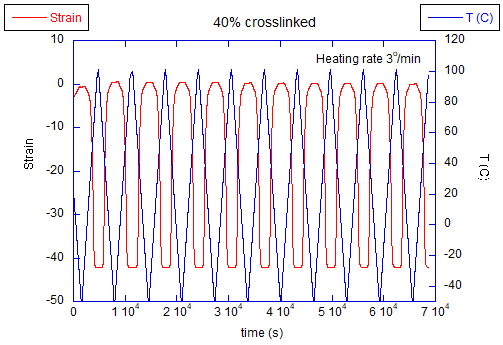
**Figure S4.** Demonstration of simple-exponential fitting of relaxation curves. Plotted on the log-linear scale, the straight-line is a clear indication of the dependence, and it is also clear that at the very late stages (when the stress falls well below 10%) there are deviations. These are caused by other factors acting in the polymer network: slow chain disentanglement, collective mesh re-arrangement, etc. However, our core information is obtained in the main region of relaxation and returns the single value of relaxation time for each material and temperature.

**Figure S5.** The detailed trace of multiple heating and cooling cycles, with the associated reversible contraction-expansion of the uniaxially aligned xLCE.
